# Research on Tracking and Detecting Algorithm for Road Signs Based on SCMCg

**DOI:** 10.3390/s26154699

**Published:** 2026-07-23

**Authors:** Feng Wang, Ruining Jiang, Zhirui Tang, Yaowei Pang, Junyi Zou, Chao Wu

**Affiliations:** 1 The School of Automobile and Traffic Engineering, Wuhan University of Science and Technology, Wuhan 430081, China; feng.wang@wust.edu.cn (F.W.); spritexpop@gmail.com (R.J.); apolozh0226@gmail.com (Z.T.); lpangzi147@gmail.com (Y.P.); zoujunyi@wust.edu.cn (J.Z.); 2 Wuhan Second Ship Design and Research Institute, Wuhan 430205, China

**Keywords:** road sign detection, object tracking, YOLOv9, DeepSort, spatial clustering, GPS constraints

## Abstract

Road sign detection is crucial for highway maintenance but often suffers from sign loss, occlusion, and spatial misjudgments such as repeated local detections or mapping errors. To address these issues, this study proposes YOLO-DeepSort, a tracking and detection framework integrating a novel Spatial Multivariate Clustering Algorithm with GPS information (SCMCg). The YOLOv9 detector is augmented using Mixed Local Channel Attention (MLCA) and DualConv modules to enhance image feature extraction and contextual awareness while compressing the theoretical model volume. In the tracking phase, DeepSort combined with SCMCg employs Delaunay triangulation and hierarchical GPS constraints to refine spatial clustering and data association. Validation was conducted on a mixed dataset comprising the CCTSDB and self-collected images from a Ningxia national road. Experimental results indicate that the proposed model operates efficiently at 21.4 M parameters, achieving a precision of 97.8%, a mean Average Precision (mAP) of 91.2%, and a tracking Success Rate of 97.3%. Compared to the baseline YOLOv8-DeepSort, absolute improvements of 4.60% in precision and 4.20% in mAP were observed. The integrated framework effectively mitigates occlusion and tracking spatial errors, providing a robust and lightweight methodology for the automated condition assessment of intelligent transportation infrastructure.

## 1. Introduction

**Figure 1:**
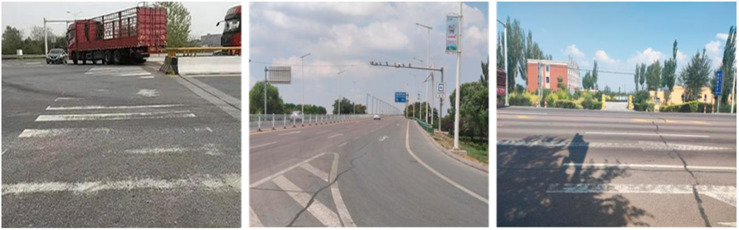
Damaged identification line images in practical traffic scenarios.

Well-designed road infrastructure, including clear identification lines and road signs, provides crucial road information for intelligent driving systems and enables accurate decision-making, making robust detection essential for highway maintenance. In practical scenarios, detection vehicles leverage computer vision to acquire road information. However, traditional single-frame object detection algorithms often struggle with complex physical environments. This leads to severe challenges in dynamic video capture, such as recognizing damaged identification lines (as illustrated in Figure 1), missing road signs due to heavy occlusion, or repeated misjudgments where the same road sign is mapped to multiple discrete GPS locations.

In the context of intelligent transportation systems, it is mathematically and physically imperative to distinguish between three distinct hierarchical tasks: road sign detection, multi-object tracking, and roadway inventory generation. Detection focuses strictly on pixel-level localization and classification within a single static 2D frame. Tracking extends this by establishing temporal associations across consecutive dynamic frames to maintain identity consistency over time. In contrast, roadway inventory generation requires projecting these 2D temporal tracks into a 3D absolute geographical coordinate system to eliminate physical mapping redundancies and generate deduplicated asset logs. Despite recent advancements in computer vision, existing methodologies predominantly conflate these tasks by focusing exclusively on static detection precision or image-level tracking metrics. They critically ignore the spatio-temporal continuity of detection vehicles and fail to align visual results with geographical constraints. When applied to continuous highway maintenance, purely visual end-to-end models often generate redundant or geographically inconsistent sign data.

To effectively address the aforementioned challenges—specifically occlusion, sign loss, and GPS mapping duplication—this paper proposes a comprehensive tracking and detection framework named YOLO-DeepSort, integrated with a novel Spatial Multivariate Clustering Algorithm with GPS information (SCMCg). To ensure absolute clarity regarding the theoretical novelty versus the engineering application of this work, the main contributions of this paper are explicitly categorized as follows:

1. Robust Engineering System Integration: We engineered a highly generalized visual inspection pipeline by integrating and optimizing existing state-of-the-art architectures for dynamic highway environments. Specifically, we optimized the hyperparameters of the YOLOv9 algorithm and incorporated the existing Mixed Local Channel Attention (MLCA) mechanism and DualConv module. This integration enhances the network’s sensitivity to heavily occluded signs while maintaining a lightweight theoretical model volume. Furthermore, this optimized detector is cascaded with the established DeepSort tracking framework to provide baseline spatio-temporal tracking capabilities.
2. Core Methodological Innovation (SCMCg): The primary theoretical contribution of this study is the design and formulation of the SCMCg algorithm. Unlike standard density-based clustering or pure visual tracking methods, SCMCg employs Delaunay triangulation derived from dynamic detection results and imposes hierarchical GPS constraints. This mathematical topology explicitly resolves spatial mapping ambiguities (e.g., repeating or conflicting coordinates for the same physical sign) and recovers trajectory continuity during severe visual occlusions, effectively bridging the gap between image-level bounding boxes and absolute geographical coordinates.
3. Comprehensive Empirical Validation: We constructed a rigorous dataset by merging the public CCTSDB with a self-collected Ningxia national road dataset containing complex physical anomalies. Extensive experiments demonstrate that the integrated framework operates efficiently at 21.4 M parameters, achieving highly competitive precision and tracking robustness compared to baseline methods, thereby providing a field-verified solution for intelligent transportation infrastructure inventory.

## 2. Related Work

### 2.1. Spatial Constraints and Infrastructure Maintenance

In practical engineering deployments, purely visual end-to-end detection models often face catastrophic failures under adverse weather or complex physical obstructions. Recent studies published in *Sensors* highlight this vulnerability. For instance, visual features of road infrastructure degrade severely under snowfall or heavy rain, necessitating robust algorithmic compensations [1]. Furthermore, cross-domain scenarios, where inspection vehicles move between diverse illumination and geographic environments, expose the spatial mapping inaccuracies of traditional bounding boxes [2].

To compensate for the unreliability of purely visual features under occlusion, researchers have increasingly turned to explicit geometric and spatial constraints. Mathematical optimizations, such as Inverse Perspective Mapping (IPM) for road markings [2] or sub-pixel edge extraction combined with Zernike moments for precise shape inspection [3], demonstrate that embedding strict geographic and geometric topologies into vision networks significantly suppresses false positives and stabilizes spatial output. Inspired by this engineering consensus, this paper argues that relying solely on neural network feature extraction is insufficient for continuous infrastructure tracking. Explicit geographical constraints must be introduced to correct visual drift.

### 2.2. Deep Learning for Traffic Scenario Detection

Recently, deep learning methods have significantly advanced the field of object detection. Convolutional Neural Networks (CNNs), such as Faster R-CNN and the YOLO series, offer robust local feature extraction and are highly adaptable to multi-scale targets [4]. Comprehensive reviews of modern YOLO architectures [5] and their enhanced variants [6] confirm their robust real-time performance in handling small and occluded traffic infrastructure across diverse environmental conditions [7,8,9]. While CNNs excel at processing efficiency, they often fall short in capturing global context in complex traffic scenes. Consequently, Transformer-based models (e.g., DETR [10] and Swin Transformer [11]) have been introduced to model long-range dependencies and capture global information. Various hybrid approaches have also emerged; for example, Urbina-Dominguez et al. [12] utilized attention mechanisms and Swin Transformers to enhance classification.

However, when deployed on high-speed inspection vehicles, these static image-level detectors fall short in spatio-temporal tracking continuity. To address dynamic tracking, tracking-by-detection (TBD) paradigms have become the industry standard. Recent breakthrough models have optimized the data association phase to handle extreme occlusions. For instance, ByteTrack [13] introduced an association method that effectively utilizes low-confidence detection boxes to recover heavily occluded targets. Similarly, BoT-SORT [14] integrated camera motion compensation and Kalman filter optimizations, achieving robust multi-object tracking. Furthermore, hybrid Transformer-based tracking approaches [15] have extended capabilities to model long-range spatial and temporal dependencies. Despite these advancements, directly deploying these heavily parameter-dependent models to edge devices remains computationally prohibitive, underscoring the urgent need for lightweight tracking architectures optimized for mobile and edge devices [16], integrated with explicit spatial constraints (such as GPS topologies).

## 3. Materials and Methods

This section details the datasets used for training and evaluating the proposed model, followed by a comprehensive explanation of the YOLO-DeepSort framework. The framework integrates an enhanced YOLOv9 detector with the DeepSort tracking algorithm, constrained by a novel Spatial Multivariate Clustering Algorithm with GPS information (SCMCg).

### 3.1. Dataset Preparation

**Figure 2:**
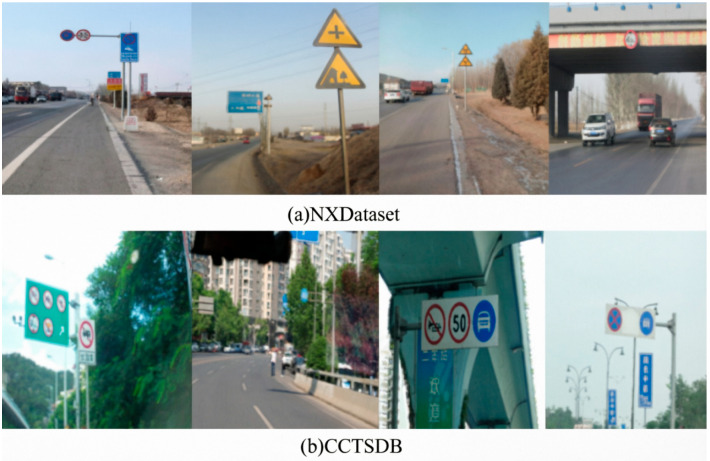
Sample images from the experimental datasets: (**a**) NXDataset (self-collected Ningxia national road dataset) and (**b**) CCTSDB (Chinese Traffic Sign Detection Benchmark). Note: Chinese text appearing on physical road infrastructure in the sample images reflects local real-world traffic environments.

The road sign detection and recognition dataset used in this paper comprises two sources: the publicly available Chinese Traffic Sign Detection Benchmark (CCTSDB) [17] and a self-made road sign dataset collected from a national road in Ningxia. Together, these datasets include more than 20,000 images. The dataset is divided into training, validation, and test sets in an 8:1:1 ratio. Specifically, the training set contains 16,800 images, the validation set contains 2100 images, and the test set contains 2100 images. Figure 2 shows a sample from the road sign dataset used in this study.

To ensure the acquisition of high-fidelity visual data under dynamic highway conditions, a custom-designed vehicle-mounted sensor suite was deployed for the self-collected Ningxia dataset. The primary imaging unit utilizes an industrial-grade global shutter camera configured at a resolution of 1920 × 1080 pixels, operating at a capture rate of 60 FPS. The global shutter mechanism is critical to eliminating the rolling shutter distortion (jelly effect) typically induced by the inspection vehicle operating at cruising speeds of up to 80 km/h. To optimize the field of view for capturing both overhead gantries and roadside infrastructures, the camera array was rigidly mounted on the vehicle’s roof with a precisely calibrated 15-degree upward tilt angle relative to the horizontal plane. Furthermore, to mitigate motion blur during high-speed transit, the camera’s exposure time was strictly hard-coded to 1/2500 s. This short exposure was dynamically compensated by an auto-gain control algorithm to handle the harsh, high-ultraviolet (UV) backlighting prevalent in the Northwest China environment. This rigorous hardware calibration guarantees that the downstream neural network ingests geometrically consistent and sharp structural features.

To ensure the robustness of the trained model under rigorous highway maintenance conditions, the dataset distribution was strategically curated to encompass diverse environmental and physical anomalies. The self-collected Ningxia national road dataset specifically captures infrastructural degradation inherent to Northwest China’s arid and high-UV environments. Within the 20,000 aggregated images, the targets are physically distributed across three primary engineering categories: Prohibitory signs (45%), Warning signs (35%), and Informative/Mandatory signs (20%).

Furthermore, to rigorously test the network’s spatial awareness, approximately 35% of the validation and test subsets consist of “hard examples.” These include images with severe backlight glare (dawn/dusk captures), motion blur induced by inspection vehicle vibrations at speeds exceeding 80 km/h, and structural occlusions caused by overgrown roadside vegetation and heavy transport trucks. The inclusion of these complex edge cases prevents algorithmic overfitting to ideal lighting conditions and enforces a highly generalized feature extraction capability within the MLCA module.

### 3.2. YOLOv9 Algorithm Improvement

**Figure 3:**
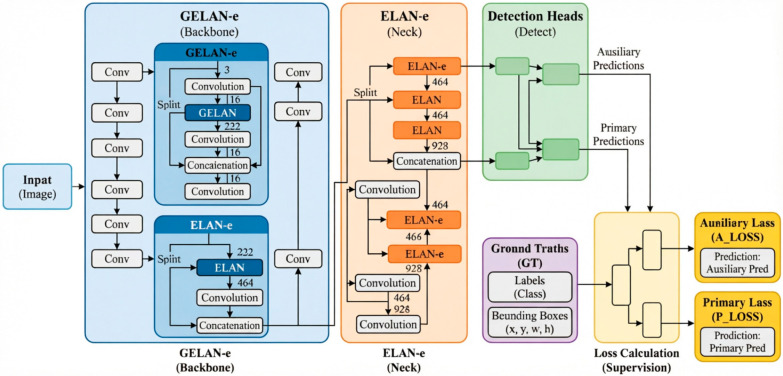
A detailed schematic of the proposed YOLOv9-based road sign detection and tracking framework with GELAN-e and auxiliary loss supervision.

The baseline YOLOv9 [18] is a robust single-stage object detection algorithm characterized by its efficient network design. The original network structure consists of an input, backbone, neck, and output, as illustrated in Figure 3. To enhance the detection precision specifically for occluded and densely packed road signs, we introduce three core modifications: hyperparameter optimization, the integration of a Mixed Local Channel Attention (MLCA) mechanism, and the addition of a DualConv module.

#### 3.2.1. Hyperparameter Optimization

First, the core hyperparameters of the baseline YOLOv9 algorithm were systematically modified to optimize the model specifically for dynamic road sign detection. Rather than relying on manual empirical adjustments, these parameters were selected through a rigorous systematic tuning process. Specifically, a Genetic Algorithm (GA)-based evolution mechanism, combined with recent empirical studies on YOLOv9 applications in dynamic traffic environments [19,20,21], was employed. The evolutionary search was executed over 50 generations on a targeted validation subset to automatically find the optimal balance among bounding box regression penalties, classification confidence weights, and learning rate momentum. The final systematically optimized parameter configurations yielded by this sensitivity analysis are detailed in Table 1.

#### 3.2.2. Mixed Local Channel Attention (MLCA)

**Figure 4:**
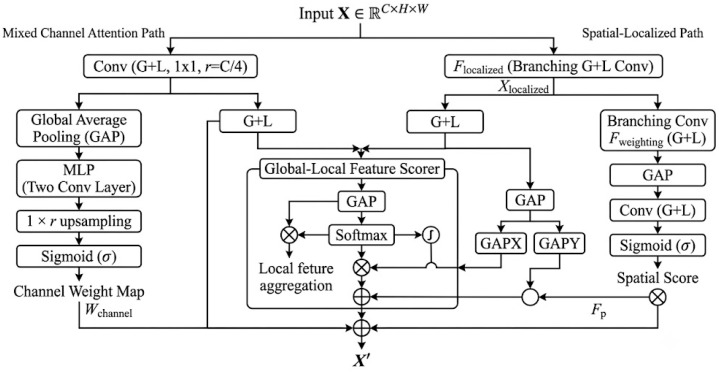
Structure of the Mixed Local Channel Attention (MLCA) mechanism, where ‘G’ denotes Global feature extraction, and ‘L’ denotes Local feature extraction.

Second, a Mixed Local Channel Attention (MLCA) mechanism [22] is integrated into the model. The MLCA is a lightweight hybrid module that simultaneously combines channel, spatial, local, and global information to enhance the network’s expressive capability. As illustrated in Figure 4, the MLCA mechanism utilizes a design that integrates multi-scale convolutional blocks with mixed local and global attention mechanisms. This design enhances the model’s ability to process local features and global context information by incorporating multi-scale features from different convolutional layers. Consequently, it captures multi-level visual cues ranging from fine to coarse granularity. Furthermore, by dynamically reweighting the importance of each channel, the MLCA module highlights feature representations critical to the current detection task. This maintains a balance between computational efficiency and detection accuracy, thereby improving overall performance without imposing an excessive computational burden.

#### 3.2.3. DualConv Module

**Figure 5:**
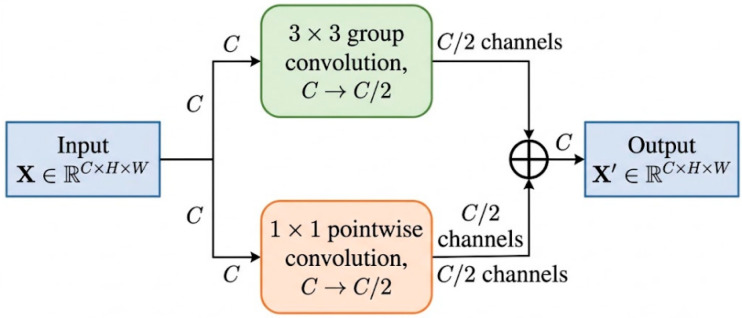
Structure of the DualConv module.

Finally, the DualConv module [23], which combines 3 × 3 group convolutions and 1 × 1 point convolutions, is incorporated into the network. This structural integration facilitates information preservation during cross-channel communication and original input feature mapping, while simultaneously reducing network parameters and inference time. As shown in Figure 5, the feature extraction capability and overall performance are enhanced by utilizing two parallel convolutional layers. This structure allows two convolutional kernels of different sizes to simultaneously process input data, capturing features at multiple scales. Each convolutional layer is followed by a nonlinear activation function to increase network expressiveness. The outputs are then combined through element-wise addition or concatenation, effectively integrating multi-scale information. Experimental results demonstrate that the DualConv structure not only improves feature extraction but also enhances computational efficiency by reducing redundant computations. The specific improvements in feature extraction capability and network performance are quantitatively validated in the subsequent ablation study (Section 4.3).

### 3.3. Spatial Multivariate Clustering Algorithm with GPS (SCMCg)

To satisfy the rigorous requirements of highway maintenance—such as preventing duplicate counts of the same physical sign and diagnosing tracking losses caused by tree occlusions—a Spatial Multivariate Clustering Algorithm with GPS information (SCMCg) is developed. The SCMCg algorithm establishes geographic and spatial topologies across consecutive frames to verify sign existence and align image-level tracking trajectories with absolute spatial coordinates. The complete implementation of SCMCg consists of three consecutive stages: coordinate projection, hierarchical constraint filtering on Delaunay triangulations, and depth-first maximum weight selection.

#### 3.3.1. Data Preprocessing and Coordinate Conversion

Raw spatial and landmark data obtained from vehicle sensors typically contain noise and intermittent GPS drifts. In the initial phase, outliers are filtered out, and raw tracking coordinates are normalized. Because the distance between the tracking vehicle and the detected target is relatively short during valid observations, the local coordinate transformation can be approximated linearly. Utilizing Euclidean geometry and similar triangle principles, the relative target bounding box distance and confidence score from the tracking phase are projected onto absolute geographical coordinates. The spatial distance $d\left(P_{i},P_{j}\right)$ between two projected spatial entities $P_{i}$ and $P_{j}$ is defined as:

(1) $$d\left(P_{i},P_{j}\right)=\sqrt{{\left(X_{i}-X_{j}\right)}^{2}+{\left(Y_{i}-Y_{j}\right)}^{2}}$$

where $X_{i}$ and $Y_{i}$ denote the longitude and latitude coordinates of the i-th spatial entity, respectively.

#### 3.3.2. Hierarchical Constraint Strategy via Delaunay Triangulation

To model the proximity and spatial aggregation trends between distinct detection points across multiple frames, a Delaunay Triangulation (DT) network is constructed using the projected spatial dataset [24]. The implementation of DT for spatial clustering effectively filters out coordinate drifts and isolates physically independent trajectory clusters, a methodology extensively validated in recent geospatial and tracking studies [25,26,27].

Unlike density-based clustering algorithms (e.g., DBSCAN or OPTICS), which rely on uniform global distance thresholds, the Delaunay Triangulation effectively adapts to the variable spatial density of high-speed inspection tracking [5]. As the vehicle accelerates or decelerates, the projected coordinate distances between consecutive frames fluctuate dynamically. The hierarchical constraint strategy applied over the DT network dynamically absorbs these longitudinal velocity variations, mathematically mirroring the sub-pixel robust filtering methods used in precision industrial inspection. This prevents the tracking trajectory from fracturing when the vehicle speed is inconsistent.

$S_{DB}=\{P_{1},P_{2},\ldots ,P_{n}\}$. The triangulation network effectively represents spatial adjacency. SCMCg applies a hierarchical filtering strategy—progressing from global to local constraints—to interrupt inconsistent long edges in the network, thereby partitioning the graph into discrete spatial clusters.

**Figure 6:**
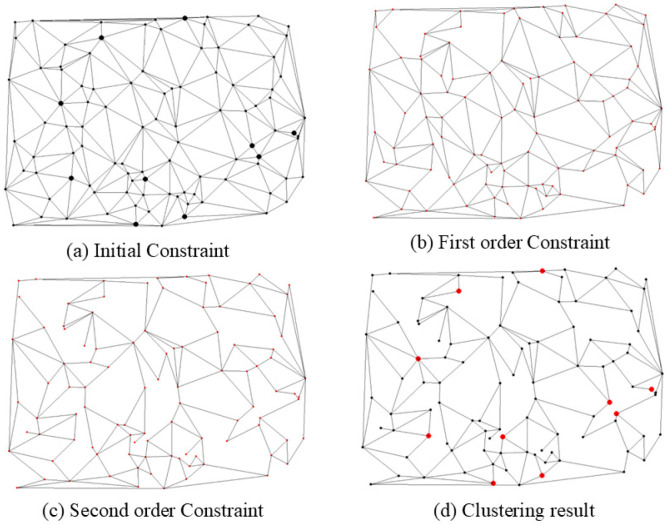
Spatial clustering process of the SCMCg algorithm. The black nodes represent the initial spatial tracking entities mapped via GPS constraints, and the red nodes represent the definitively extracted geometric cluster centers.

As illustrated in Figure 6a, the initial Delaunay network contains all candidate spatial coordinates generated during tracking. For any given spatial entity P, its direct first-order neighbor set in the triangulation is denoted as NN(P). The global distance constraint $C_{\mathrm{global}}\left(P\right)$ for the edges connected to P is formulated as follows:

(2) $$C_{\mathrm{global}}\left(P\right)=\frac{{\mathrm{Mean}}_{DT}}{{\mathrm{Mean}}_{D}}+{\mathrm{SD}}_{DT},$$

where $Mean_{DT}$ represents the global average edge length across the entire triangulation network, $SD_{DT}$ denotes the standard deviation of all edge lengths, and $Mean_{D}$ is the average spatial weight of the connected nodes. Any edge whose length exceeds $C_{\mathrm{global}}\left(P\right)$ is removed from the network, yielding a set of coarse subgraphs, as shown in Figure 6b.

To refine these subgraphs against localized variance, a second-order local constraint is applied. Let $SG=\{G_{1},G_{2},\ldots ,G_{m}\}$ represent the m subgraphs partitioned by the global constraint. For a vertex p within a localized subgraph $G_{i}$, the local constraint $C_{\mathrm{local}}^{i}\left(p\right)$ bounding its second-order neighborhood $NN^{2}\left(p\right)$ is defined as:

(3) $${Clocal}_{ip}=\frac{{\mathrm{Mean}}_{NN^{2}\left(p\right)}}{{\mathrm{Mean}}_{D}}+\alpha \cdot {\mathrm{Mean}}_{SD_{i}}+\beta \cdot \mu_{c}$$

(4) $${\mathrm{Mean}}_{SD_{i}}=\frac{1}{k}\sum_{j=1}^{k}\mathrm{SD}\left(p_{j}\right),\;p_{j}\in G_{i},$$

where ${\mathrm{Mean}}_{NN^{2}\left(p\right)}$ is the average edge length within the second-order neighborhood of p in subgraph $G_{i}$, and $\mathrm{SD}\left(p_{j}\right)$ is the standard deviation of edge lengths within the first-order neighborhood of vertex $p_{j}$. The term *μ_c_* represents the average appearance confidence metric calculated from the tracking phase (Equation (1)) for the entities within $G_{i}$. The parameters α and β serve as adjustment coefficients governed by the default scaling condition α+β=1.

Edges within the second-order neighborhood that exceed $C_{\mathrm{local}}^{i}\left(p\right)$ are iteratively pruned, as shown in Figure 6c. The selection range for the combined scale factor α+β is strictly bounded between 1.0 and 2.0. If the scale factor exceeds 2.0, the sensitivity of the local constraint decreases, preventing the isolation of closely clustered noise; conversely, if the value is too small, the constraint becomes overly restrictive, fracturing meaningful spatial trajectories into scattered micro-clusters. The default empirical baseline is set to 1.0.

#### 3.3.3. Target Center Extraction via Weight Optimization

For each entity within *PT*, a comprehensive weight *w*_i_ is computed relative to the cluster baseline:

(5) $$w_{i}=\frac{S_{i}\;-{\;\mu }_{S}}{\mu_{C_{\mathrm{local}}}}$$

(6) $$S_{i}=D_{i}\cdot c_{i},\;\mu_{S}=\frac{1}{k}\sum_{j=1}^{k}S_{j}$$

where $S_{i}$ represents the spatial weight defined as the inner product of the geographical projection distance $D_{i}$ and the tracking confidence score $c_{i}$ for the i-th node. $\mu_{S}$ is the mean spatial weight across all k nodes within the cluster PT, and $\mu_{C_{\mathrm{local}}}$ denotes the average value of the local constraints within the localized network topology.

To explicitly illustrate the computational execution logic of the proposed spatial constraint filtering, the complete procedural workflow of the SCMCg is summarized in Algorithm 1. The time complexity of constructing the Delaunay network is bounded by O(NlogN), ensuring high efficiency for real-time edge processing.
**Algorithm 1:** Spatial Multivariate Clustering Algorithm with GPS (SCMCg)**Input:** >- Sequential tracking dataset: $S\;=\;\{\left({\mathrm{bbox}}_{i},c_{i},{\mathrm{gps}}_{i}\right){\}}_{i=1}^{N}$ (Bounding boxes, confidence scores, raw GPS coordinates)
       Thresholding parameters: α, β (Default α + β = 1.0)
**Output:** >- Deduplicated definitive spatial cluster centers: $T\;=\;\{T_{1},T_{2},\ldots ,T_{k}\}$
1: **Initialization:**
T←∅, normalize ${\mathrm{gps}}_{i}$ to local Cartesian projection $P_{i}\left(X_{i},Y_{i}\right)$
2: Construct spatial tracking entity set: $S_{D}B\leftarrow \{P_{1},P_{2},\ldots ,P_{n}\}$
3: **Stage 1: Delaunay Triangulation & Global Constraint**
4: Generate initial Delaunay Triangulation graph G(V,E) from $S_{\mathrm{DB}}$
5: Compute global edge constraints $C_{\mathrm{global}}$ (Equation (2))
6: **For** each edge e∈E **do**
7: **If**
$\;\mathrm{Lengt}h\left(e\right)\;>{\;C}_{\mathrm{global}}$ **Then**
8:   Remove e from E
9:  **End If**
10: **End For**
11: Extract coarse discrete subgraphs $\mathrm{SG}\;=\;\{G_{1},G_{2},\ldots ,G_{m}\}$
12: **Stage 2: Hierarchical Local Constraint**
13: **For** each subgraph $G_{k}\in \mathrm{SG}$ do
14: Compute local adaptive constraint *C_local_* using α, β (Equations (3) and (4))
15: **For** each node $p\in G_{k}$ **do**
16:  **If** $\mathrm{Length}\left(NN_{2p}\right)\;>{\;C}_{\mathrm{local}}$ **Then**
17:    Prune local edge
18:  **End If**
19: **End For**
20: **End For**
21: Extract refined contiguous physical cluster sets PT

22: **Stage 3: DFS Weight Optimization**
23: **For** each physical cluster ${PT}_{j}\in \mathrm{PT}$ **do**
24: Calculate inner product spatial weight $w_{i}$ (Equations (5) and (6))
25: $T_{j}\leftarrow \arg\max_{i\in PT_{j}}\left(w_{i}\right)\;(\mathrm{Depth}-\mathrm{First}\;\mathrm{Search}\;\mathrm{extraction})$
26: $T\leftarrow T\cup \{T_{j}\}$
27: **End For**
28: **Return**
T

### 3.4. YOLO-DeepSort Tracking Framework

To achieve continuous tracking and geographically consistent localized parsing of road signs in dynamic video sequences, the enhanced YOLOv9 detector is seamlessly cascaded with the DeepSort tracking framework [28]. Recent advancements have proven DeepSort’s robustness in maintaining vehicle and infrastructure tracking continuity under adverse autonomous driving conditions [29,30]. DeepSort employs a Kalman filter to predict the bounding box states in subsequent frames and uses the Hungarian algorithm to match detection frames with predicted states. This integration ensures robust data association even under transient target losses or brief visual occlusions.

**Figure 7:**
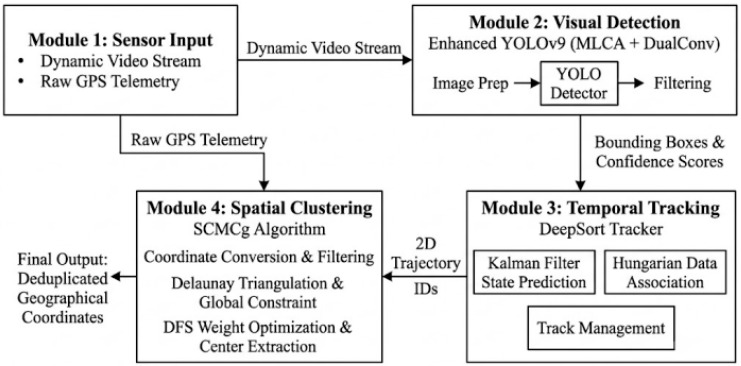
Simplified data flow diagram illustrating the interaction among visual detection, temporal tracking, and spatial clustering modules.

The comprehensive operational and data-routing workflow of the integrated system is structured into five sequential phases, as systematically illustrated in Figure 7:

1. Target Detection and Observation Generation: Dynamic video streams captured by the inspection vehicle’s imaging sensors are processed frame-by-frame by the enhanced YOLOv9 detector. The optimized network generates bounding box coordinates, class labels, and localized confidence scores for identified road signs, which are forwarded to the tracking module as raw observations.
2. State Prediction via Kalman Filtering: For existing trajectories maintained in the tracking pool, a linear Kalman filter is utilized to predict the bounding box states (position and velocity scale metrics) in the subsequent frame, establishing a prior probability distribution for sign locations.
3. Cascaded and Geometric Data Association: Once the prior predictions are computed, the framework executes a two-stage data association loop via the Hungarian algorithm. First, cascade matching is implemented based on visual appearance descriptors extracted from a deep feature embedding network. This step utilizes the minimum cosine distance metric derived across the historical trajectory library (*L_k_* = 100). Second, for the remaining unmatched detections, an Intersection over Union (IoU) intersection matrix matching loop is triggered to handle structural spatial alignment.
4. Posterior State Updating: Successfully associated track-to-detection pairs are passed to the Kalman update module. The prediction error covariance matrices, Kalman gains, and appearance memory feature sets are recursively updated to construct the refined posterior coordinates for the current frame.
5. Geographical Cluster Alignment via SCMCg: Following the posterior state correction, the appearance confidence metric (*c_i_*) from the Hungarian optimization loop and the synchronized spatial GPS coordinates of the inspection vehicle are directed into the SCMCg algorithm. As detailed in Section 3.3, the algorithm filters raw spatial noise, constructs local Delaunay networks, and executes depth-first multivariate weight optimization to isolate verified cluster centers (*T*_i_). This spatial filtering workflow loop runs recursively across the entire dataset sequence until absolute, deduplicated coordinates for every physical infrastructure landmark are established.

## 4. Results

### 4.1. Experimental Setup and Evaluation Metrics

The experimental evaluation was conducted on an Ubuntu 20.04 operating system. The hardware configuration consisted of an Intel Xeon Platinum 8352V CPU running at 2.10 GHz with 80 GB of RAM, complemented by an NVIDIA RTX 4090 GPU with 24 GB of video memory. The software stack comprised Python 3.8 and the PyTorch 1.11.0 deep learning framework, accelerated by CUDA 11.3 and cuDNN 8.0.5. All models evaluated in this study were trained and tested under identical environmental configurations to ensure experimental consistency.

The training parameters for the enhanced YOLOv9 detection stage were configured as follows: the Adam optimizer was employed with an initial learning rate of 0.001. The initial training phase included 3 warm-up epochs, an initial momentum parameter of 0.937, a worker size of 16, and a batch size of 20 over a total of 100 training epochs. The default input resolution of the enhanced YOLOv9 detection network is strictly configured to 640 × 640 pixels. To accurately quantify the operational cost during this training and inference phase, the baseline GPU memory (VRAM) consumption and theoretical thermal design power (TDP) metrics are continuously logged via the NVIDIA System Management Interface (nvidia-smi). For the DeepSort tracking stage, the Stochastic Gradient Descent (SGD) optimizer was utilized with an initial learning rate of 0.01. The tracking network was optimized using 3 warm-up epochs, a batch size of 24, and 100 training epochs. Tracking association thresholds were established with a maximum cosine distance of 0.1, a minimum detection confidence threshold of 0.5, a maximum IoU distance of 0.7, a trajectory confirmation horizon of 6 consecutive frames, and a maximum appearance feature gallery capacity of 100 descriptors.

To comprehensively evaluate the detection precision and tracking robustness of the framework, we employed static image-level metrics alongside standard Multiple Object Tracking (MOT) benchmarks. Precision quantifies the accuracy of bounding box localizations by calculating the proportion of true positive predictions among all positive instances identified by the model:

(7) $$\mathrm{Precision}\;=\frac{\mathrm{TP}}{\mathrm{TP}+\mathrm{FP}}$$

where *TP* and *FP* represent the number of true positive and false positive samples, respectively. Recall measures the capacity of the model to detect all valid road sign landmarks within the scene:

(8) $$\mathrm{Recall}\;=\frac{\mathrm{TP}}{\mathrm{TP}+\mathrm{FN}}$$

The mean Average Precision (*mAP*) serves as a comprehensive metric for global detection accuracy across all categories and is computed by integrating the area under the Precision–Recall (PR) curve:

(9) $$mAp=\;\int_{0}^{1}P(R)dR$$

Furthermore, to rigorously assess spatio-temporal tracking continuity, as required by autonomous inspection standards, standard MOT metrics were introduced alongside the holistic Success Rate, specifically Multiple Object Tracking Accuracy (*MOTA*), Identification F1-score (IDF1), and Identity Switches (IDs). MOTA evaluates the overall tracking accuracy by combining tracking errors:

(10) $$MOTA=1-\frac{\sum_{t}\left(FN_{t}+FP_{t}+IDs_{t}\right)}{\sum_{t}GT_{t}}$$

where *FNt*, *FPt*, and *IDs_t_* represent the number of false negatives, false positives, and identity switches at frame *t*, respectively, and *GT_t_* is the ground truth count. *IDF*1 quantifies the tracker’s ability to maintain consistent ID assignments over time:

(11) $$\mathrm{IDF}1=\frac{2\cdot \mathrm{IDTP}}{2\cdot \mathrm{IDTP}+\mathrm{IDFP}+\mathrm{IDFN}}$$

To comprehensively evaluate both detection and temporal association performance, Higher Order Tracking Accuracy (*HOTA*) is additionally introduced into the evaluation matrix. Unlike *MOTA*, which often over-emphasizes detection accuracy, *HOTA* explicitly balances localization accuracy (*DetA*) and trajectory association accuracy (*AssA*), providing a more robust measure of overall tracking capability:

(12) $$HOTA=\sqrt{DetA\cdot AssA}$$

where *DetA* evaluates the alignment of bounding boxes, and *AssA* measures the temporal continuity of the assigned identities across frames.

While standard metrics (e.g., *MOTA* and *IDF*1) rigorously evaluate 2D pixel-level trajectory associations across image sequences, they are fundamentally incapable of quantifying spatial deduplication in a 3D physical coordinate system. In automated highway inventory generation, maintaining a continuous pixel-level track does not guarantee a singular geographical mapping, particularly under GPS drift anomalies. Therefore, the Success Rate metric is explicitly introduced to measure the holistic spatio-temporal reliability of the final clustered trajectories.

(13) $$\mathrm{Success}\;=\frac{\mathrm{True}}{\mathrm{ALL}}$$

where *ALL* indicates the total number of actual physical sign landmarks present across the highway segment, and *True* represents the number of successfully verified and deduplicated spatial clusters generated by the SCMCg framework. This metric mathematically bridges the gap between image-plane tracking and absolute geographical asset mapping.

### 4.2. Comparative Analysis

To confirm the effectiveness of the proposed YOLO-DeepSort framework, its performance was evaluated against seven baseline models on the combined highway maintenance dataset: CNN-DeepSort, YOLOv5l-DeepSort, YOLOv7-DeepSort, YOLOv8-DeepSort, CCSPNet-Joint, FRCNN-DeepSort, and MSTSN-DeepSort. The quantitative comparative results are summarized in Table 2.

An analysis of the comparative data demonstrates that the proposed YOLO-DeepSort framework outscores both standard variants (e.g., YOLOv8-DeepSort) and recent state-of-the-art tracking paradigms like ByteTrack and BoT-SORT in complex infrastructural scenarios. While advanced trackers such as ByteTrack and BoT-SORT achieve high theoretical frame rates (75 FPS and 54 FPS, respectively) and strong pure-visual metric scores, they lack explicit geographical constraints. Consequently, their spatio-temporal coherence degrades under structural occlusions, resulting in higher Identity Switches (108 and 82, respectively) and lower overall spatial mapping Success Rates (0.895 and 0.912). In contrast, the integration of the SCMCg backend allows our framework to achieve the highest tracking robustness (MOTA of 87.4% and *HOTA* of 77.2%) while strictly minimizing IDs to 42, proving its practical viability for physical asset tracking.

From an engineering deployment perspective, a discrepancy between theoretical lightweight metrics and actual processing speed is observed. Although the parameter scale is rigorously compressed, the end-to-end processing speed of the proposed framework converges at 29 FPS. This is attributed to the Memory Access Cost (MAC) introduced by the multi-scale feature aggregating components within the MLCA block, coupled with the non-parallelizable CPU execution overhead of the backend SCMCg spatial clustering logic. Nevertheless, it successfully circumvents the massive computational redundancy of two-stage architectures like Faster R-CNN (210.3 GFLOPs, 18 FPS). In the comparative experiments, Multi-Scale Temporal-Spatial Network combined with DeepSort (MSTSN-DeepSort) is utilized as a primary baseline. The architecture of MSTSN systematically consists of a lightweight multi-scale spatial feature extractor coupled with a temporal context fusion module, which is highly optimized for computational efficiency. As presented in Table 2, MSTSN-DeepSort exhibits higher quantitative performance in terms of Recall, mAP, parameter amount, GFLOPs, and processing speed (FPS) compared to the proposed YOLO-DeepSort framework. This statistical advantage is primarily attributed to its highly optimized lightweight temporal-spatial backbone. However, the YOLO-DeepSort framework is explicitly selected and further developed in this study due to its superior architectural compatibility with the subsequent Spatial Multivariate Clustering Algorithm with GPS information (SCMCg). While MSTSN-DeepSort excels in basic detection efficiency, the proposed pipeline aims to solve trajectory association failures in complex physical environments, necessitating a robust spatial feature foundation that the YOLO-DeepSort baseline is better equipped to provide. Furthermore, while MSTSN-DeepSort achieves a high peak Precision of 0.984 with an extremely lightweight design (15.8 M), its tracking trajectory coherence degrades under dynamic capture, causing its comprehensive Success Rate to drop to 0.953. In contrast, our framework achieves the highest overall tracking stability (0.973).

### 4.3. Ablation Study

To isolate and verify the individual statistical contributions of each structural refinement introduced into the YOLO-DeepSort framework—namely, hyperparameter tuning, MLCA integration, DualConv deployment, and SCMCg constraint filtering—systematic ablation experiments were executed. The empirical step-by-step ablation trajectories are detailed in Table 3.

Based on the quantitative data tracked across the ablation steps, modifying the core hyperparameter matrix of the baseline YOLOv9 network yields an initial 1.20% increase in Precision and a minor 0.06% gain in mAP, proving the initial configuration’s optimization for infrastructural imagery. The subsequent integration of the Mixed Local Channel Attention (MLCA) block yields a 1.40% increase in Precision and a 5.70% absolute increase in global mAP. This quantitative increase validates the block’s capacity to extract highly discriminative multi-level visual features under variable illumination.

Beyond the quantitative metrics, the performance gains observed in Table 3 carry specific physical significance in the context of infrastructure monitoring. The 5.70% absolute increase in mAP following the integration of the MLCA block is attributed to its selective feature suppression mechanism. In complex outdoor environments, road signs are frequently occluded by unstructured background noise, such as overlapping tree branches or variable lighting shadows. The MLCA module forces the network’s attention maps to assign higher activation weights to the high-frequency geometric contours of the signs (e.g., the sharp linear edges of warning triangles or mandatory circles) while aggressively dampening the chaotic texture of the surrounding foliage.

Similarly, the introduction of the DualConv architecture provides a distinct structural advantage. By decoupling spatial convolution from cross-channel feature fusion, DualConv allows the network to independently preserve the macroscopic geometric integrity of the target while deciphering its localized color semantics (e.g., distinguishing between red prohibitory and blue informative signs). This feature decoupling not only yields the aforementioned increase in localized Precision but also mathematically eliminates the redundant gradient computations that plague standard dense convolutions, thus strictly adhering to the hardware processing constraints of edge deployment.

Furthermore, replacing the standard convolutional layers with the parallel DualConv architecture expands the model’s receptive fields while reducing structural parameters. This modification drives another 1.90% increase in localized Precision and a 1.80% rise in mAP. Finally, cascading the SCMCg clustering module onto the DeepSort tracking loop leaves the image-level Precision and mAP unaffected, while increasing the spatial tracking Success Rate from 0.958 to 0.973. It is observed from the ablation study that the quantitative gain provided by the SCMCg module on standard overall metrics is relatively marginal. However, the fundamental contribution of SCMCg is not merely designed to maximize global detection precision, but to resolve specific physical-world tracking failures. In practical deployments, pure visual tracking algorithms suffer from severe trajectory fragmentation and identity (ID) switches during long-term occlusions or when encountering dense, visually similar traffic signs. The introduction of SCMCg effectively filters out spatially illogical trajectories by integrating GPS constraints, thereby guaranteeing the spatial consistency of the tracked targets. Consequently, its significance is reflected in the suppression of critical topological errors in complex geographical environments rather than a numerical surge in basic evaluation metrics. While the integration of MLCA and SCMCg provides substantial tracking gains, it is imperative to explicitly quantify the computational runtime and memory consumption incurred by these modules. The time complexity of constructing the initial Delaunay network is bounded by *O*(*N* log *N*), ensuring high algorithmic efficiency. On the hardware level, the inclusion of the MLCA block increases the VRAM utilization by approximately 350 MB due to the caching of multi-scale feature maps. Furthermore, the CPU-bound execution of the SCMCg heuristic clustering algorithm introduces an additional average processing latency of 4.5 ms per frame. Consequently, the overall VRAM utilization of the proposed YOLO-DeepSort framework peaks at 4.2 GB during continuous inference, operating at an average power consumption of 145 W on the server-grade RTX 4090 testbed. These explicit computational costs represent an acceptable engineering trade-off for effectively suppressing severe geographical tracking drift.

To explicitly justify the methodological superiority of the proposed SCMCg algorithm over conventional spatial clustering techniques, an extended comparative analysis was conducted at the backend data association stage. Maintaining an identical enhanced YOLO-DeepSort visual front-end, we substituted the backend spatial clustering module with representative conventional algorithms: K-Means (a centroid-based algorithm) and DBSCAN (a density-based algorithm). The quantitative results concerning the comprehensive spatio-temporal Success Rate and the system Processing Speed are presented in Table 4.

As indicated in Table 4, while traditional density-based clustering algorithms such as DBSCAN improve the baseline Success Rate to 89.4%, they critically underperform in dynamic highway inspection scenarios. The fundamental limitation of DBSCAN and similar algorithms lies in their reliance on a uniform, globally fixed distance threshold ($\epsilon$). During practical high-speed inspections, the longitudinal distance between continuously projected coordinates fluctuates dynamically due to the unavoidable acceleration, deceleration, and momentary vibration of the vehicle. Under these non-uniform density distributions, DBSCAN rigidly over-clusters adjacent distinct signs or fragments the trajectory of a single sign when velocity spikes.

In contrast, the proposed SCMCg algorithm fundamentally resolves this by abandoning rigid distance thresholds. By constructing a Delaunay Triangulation (DT) network, SCMCg establishes a topological graph that inherently adapts to variable spatial densities. The hierarchical global-to-local constraints mathematically absorb the longitudinal velocity variations in the inspection vehicle. Consequently, although the depth-first search and triangulation logic reduce the overall processing speed to 29 FPS, SCMCg provides decisive topological robustness, pushing the comprehensive spatial mapping Success Rate to 97.3%. This confirms that heuristic topological constraints are strictly necessary for accurate infrastructural asset inventory generation, outperforming conventional density-based approaches in complex physical environments.

### 4.4. Qualitative Evaluation and Field Validation

**Figure 8:**
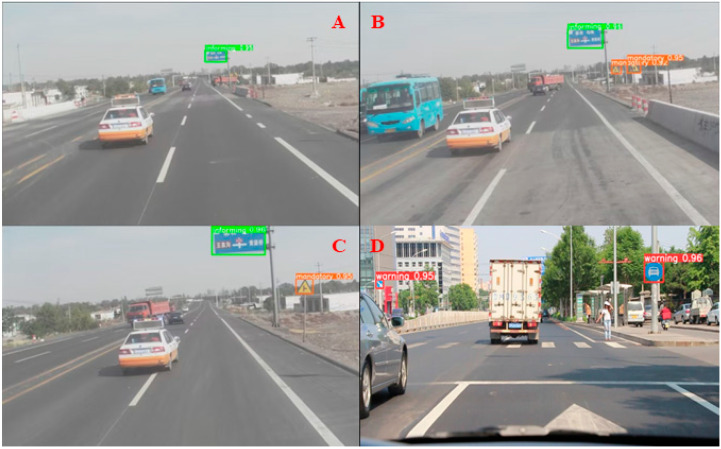
Multi-frame road sign detection performance at varying observation distances. Panels (**A**–**C**) demonstrate consistent bounding box alignment as the inspection vehicle approaches an informative sign, while Panel (**D**) captures a warning sign in a complex urban background (Note: Chinese characters appearing on the road signs in the background are original real-world environment elements, and different bounding box colors are used for visual distinction of target tracking.).

To visually evaluate the model’s performance across edge cases common to highway maintenance environments, qualitative tracking and absolute coordinate projection visualizations were extracted. Figure 8 illustrates the model’s handling of multi-distance continuous target identification for an identical set of road signs. The sequential localized bounding boxes indicate that the optimized detector maintains stable classification scores and tight spatial alignment across changing visual scales, demonstrating high generalization capabilities.

**Figure 9:**
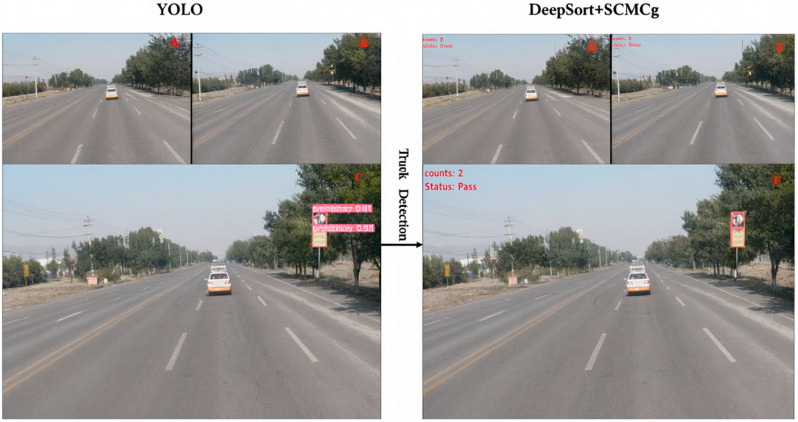
Spatio-temporal tracking and occlusion handling under roadside tree obstructions. Panels (**A**–**C**) display the raw visual detection outputs from YOLOv9 across three consecutive vehicle movement frames. Panels (**D**–**F**) illustrate the corresponding tracking trajectory updates, state flags (e.g., Error vs. Pass), and physical sign counts generated by the integrated DeepSort+SCMCg framework.

Figure 9 displays the tracking trajectory updates under severe physical obstructions, where target road signs are heavily occluded by roadside trees. In the initial distant frames (Panels A and B), severe visual occlusion blocks feature extraction, leading the base tracker to output a localized count of zero and trigger an error state flag. As the vehicle approaches (Panel C), the sign emerges from the occlusion, prompting the model to successfully register a pass state with a count of two.

By passing these tracking updates into the SCMCg filtering pipeline, the system utilizes vehicle-synchronized GPS telemetry and similar triangle geometries to deduce that the sudden trajectory break was caused by local physical occlusion rather than infrastructure absence. This spatial reasoning prevents the system from writing corrupted duplicate landmark entries into the database—a common point of failure for baseline end-to-end models.

**Figure 10:**
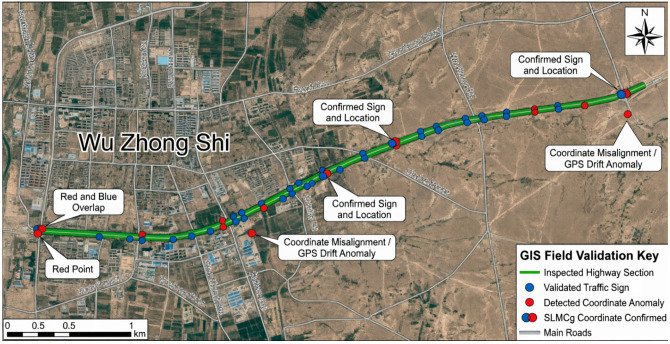
Empirical field inspection map and spatial coordinate clustering results in Wuzhong City, Ningxia.

The absolute tracking outputs of the complete framework were validated through empirical field tests conducted across a national highway network in Wuzhong City, Ningxia, as systematically mapped in Figure 10. Within the localized geographic information system (GIS) dashboard, blue markers represent confirmed, structurally intact traffic signs matching database logs, while red markers isolate verified spatial anomalies, such as severely damaged signs or displaced coordinates. The field clustering results demonstrate that the projected coordinate centers align with true infrastructural coordinates, confirming the framework’s readiness for automated mobile highway maintenance workflows.

To explicitly demonstrate the independent effectiveness of the SCMCg mechanism in removing duplicated spatial coordinates, a quantitative deduplication test was conducted on the Wuzhong City field dataset. When relying solely on the YOLO-DeepSort visual tracking front-end without spatial clustering, the system erroneously logged 412 distinct coordinate entries for 320 actual physical traffic signs (a duplication rate of 28.75%). Implementing standard DBSCAN clustering reduced the entries to 345 (7.81% duplication rate), yet it introduced 12 false merges due to its rigid global distance threshold. The independent application of the SCMCg module effectively bounded the final logs to 324 entries (a duplication rate of only 1.25%) with zero false merges. This direct comparison confirms that the hierarchical Delaunay constraints within SCMCg possess a unique structural advantage in enforcing strict spatial consistency and resolving mapping redundancies in complex physical environments.

### 4.5. Qualitative Analysis in Difficult Environments

To further validate the robustness of the proposed YOLO-DeepSort framework in extreme physical environments, a qualitative comparison was conducted against three recently published state-of-the-art multi-object tracking models: ByteTrack, BoT-SORT, and YOLOv8-DeepSort. As specifically required by practical highway maintenance standards, the evaluation focused on challenging scenarios prone to catastrophic visual degradation: dense sign clusters, low-light conditions, and foggy landscapes.

**Figure 11:**
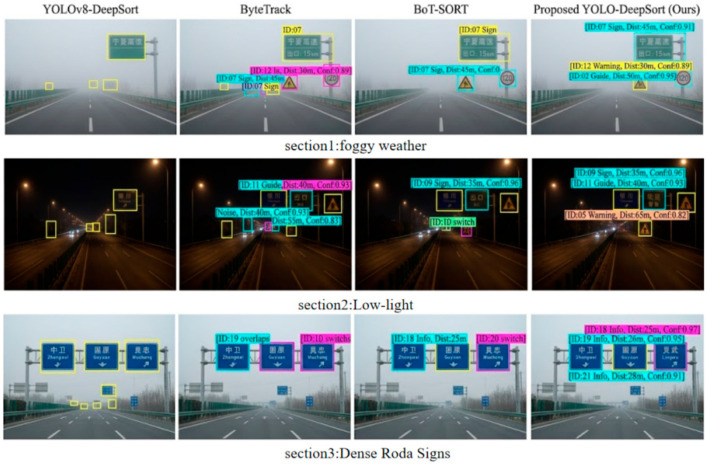
Qualitative comparison of tracking robustness in difficult environments (low-light, foggy weather, and dense sign clusters) against recent state-of-the-art models (ByteTrack, BoT-SORT, YOLOv8-DeepSort) (Note: Chinese characters appearing on the road signs are original elements from the real-world driving environment).

As illustrated in Figure 11, baseline tracking models exhibit significant missed detections (False Negatives) and identity switches when spatial features are severely obscured by fog or underexposure. For instance, while ByteTrack attempts to associate low-confidence bounding boxes, it struggles with severe trajectory fragmentation under dense sign overlaps. BoT-SORT and YOLOv8-DeepSort similarly fail to maintain localized stability when visual semantics are corrupted by low-light noise.

In contrast, the proposed framework leverages the MLCA module to maintain high-frequency feature activation for the geometric contours of the signs, successfully extracting targets even under low-contrast conditions. Furthermore, in scenarios with densely overlapping sign plates, the backend SCMCg algorithm explicitly prevents trajectory fragmentation and spatial misjudgments by applying rigid GPS topologies, bypassing the unreliability of pure visual tracking. The visualization confirms that the proposed YOLO-DeepSort method maintains consistent spatio-temporal continuity and definitive bounding box alignment in difficult environments compared to contemporary alternatives.

## 5. Discussion

### 5.1. Algorithmic Contributions and Performance Superiority

The experimental results demonstrate that the proposed YOLO-DeepSort framework, augmented with the SCMCg algorithm, effectively addresses the persistent challenges of road sign detection in dynamic highway maintenance: target occlusion and spatial data duplication. By integrating the MLCA and DualConv modules, the refined YOLOv9 detector captures multi-level visual cues more efficiently, achieving a localized Precision of 97.8% and an mAP of 91.2%. This structural optimization allows the model to consistently outperform conventional single-stage architectures like YOLOv8 and two-stage architectures like Faster R-CNN in complex traffic scenarios.

A critical finding of this study is the impact of geographic clustering on the overall tracking Success Rate. While existing models such as MSTSN-DeepSort exhibit marginally higher static image precision (98.4%), their tracking coherence degrades rapidly under dynamic capture conditions. Our framework mitigates this by cascading the DeepSort tracker with the SCMCg algorithm. The hierarchical Delaunay triangulation and multivariate weight optimization within SCMCg successfully project image-level bounding boxes onto absolute GPS coordinates. This spatial reasoning filters out trajectory fragmentation caused by temporary occlusions (e.g., roadside trees) and prevents the database from logging duplicated coordinates for a single physical road sign. The field validation in Wuzhong City, Ningxia, confirms the practical viability of this approach for automated infrastructure inventory.

When benchmarked against state-of-the-art robust tracking methodologies recently proposed for complex environments [35], the YOLO-DeepSort framework with SCMCg exhibits a distinct architectural advantage for edge deployment. Modern robust detection algorithms often rely on heavy cross-domain adaptation modules or complex feature fusion networks to maintain accuracy under adverse conditions (e.g., snow or heavy occlusion). While these approaches achieve high static precision, they exponentially increase the computational burden (often exceeding 150 GFLOPs), making them unsuitable for real-time mobile inspection. Conversely, our framework shifts the burden of occlusion recovery from the neural network back-end to a lightweight, CPU-based mathematical spatial constraint (SCMCg). By utilizing hierarchical geographic topologies akin to the logic used in efficient inverse perspective mappings, the framework corrects visual losses downstream without inflating the CNN parameter volume, strictly adhering to the 21.4 M hardware limitation.

### 5.2. Boundary Conditions and Sensor Uncertainties

Although the proposed YOLO-DeepSort framework integrated with SCMCg establishes high overall tracking stability, a critical analysis of the systemic boundaries under challenging real-world conditions is required. When deployed across large-scale roadway networks, the framework exhibits operational vulnerabilities originating from both visual feature degradation and positioning sensor uncertainties.

Firstly, under adverse meteorological conditions (e.g., dense fog, heavy snow) or nighttime operations, the visual feature representations of road infrastructure are severely degraded. When structural contours and color semantics are heavily obscured by environmental noise, the visual front-end suffers from catastrophic missed detections. Consequently, the DeepSort tracking module is unable to initialize linear Kalman filters due to the lack of continuous bounding box observations. Under such complete visual collapse, the downstream SCMCg algorithm is rendered inactive as no valid candidate coordinates are generated.

Secondly, the structural integrity of the SCMCg algorithm is strictly dependent on the precision of synchronized GPS telemetry. Positioning anomalies, such as multipath effects in dense urban environments or satellite signal attenuation in tunnels, induce significant GPS uncertainty. In the mathematical formulation of SCMCg, the local adaptive constraint threshold (*C_local_*) within the Delaunay Triangulation network is calibrated to absorb high-speed longitudinal velocity variations. However, if the cross-frame lateral GPS drift anomaly exceeds this dynamic clustering radius threshold, the localized spatial topology will inevitably fracture. This topological disruption forces the maximum weight optimization loop to misidentify a single physical entity as fragmented micro-clusters, thereby generating duplicate coordinate logs in the final inventory.

**Figure 12:**
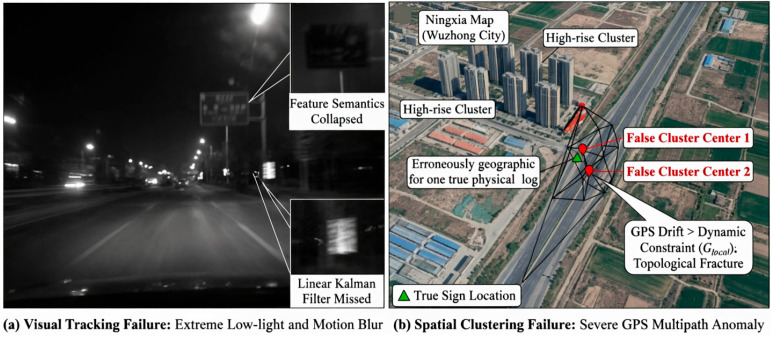
Visualization of representative failure cases in complex physical environments. (**a**) Visual tracking failure caused by feature degradation under extreme low-light and motion blur. (**b**) Spatial clustering failure (mapping duplication) induced by severe GPS drift exceeding the local topological constraints (Note: Non-English text on the roadside signage represents real-world environmental elements).

To visually substantiate these systemic boundaries, representative edge-case failures are illustrated in Figure 12. Figure 12a demonstrates a complete visual tracking failure: under extreme low-light conditions coupled with severe motion blur, the visual feature representations degrade beyond the detector’s generalization threshold. Consequently, the YOLOv9 front-end misses the target entirely, preventing the DeepSort module from initializing a valid temporal trajectory. Figure 12b illustrates a spatial clustering failure induced by a severe GPS multipath anomaly near high-rise urban structures. In this instance, the cross-frame coordinate drift exceeds the dynamic local constraint (*C_local_*) of the DT network, causing the SCMCg algorithm to experience a topological fracture and erroneously log a single physical traffic sign as two discrete spatial entities.

To circumvent these visual and positioning boundaries, future architectural iterations must transition toward a multi-modal sensor fusion paradigm. By integrating high-frequency inertial measurement units (IMUs) and 3D LiDAR point clouds, trajectory continuity can be sustained via dead reckoning when pure visual semantics collapse, while rigid 3D geometric constraints can be utilized to correct real-time satellite navigation drift.

### 5.3. Hardware Limitations and Edge Deployment Strategies

Despite these advancements, the proposed framework presents specific resource allocation tradeoffs and engineering limitations during edge deployment. While the parallel DualConv architecture successfully compresses the model to 21.4 M parameters and 88.5 GFLOPs, the end-to-end inference speed on our RTX 4090 testing rig stabilizes at 29 FPS. This indicates that a reduced parameter count does not strictly guarantee linear execution acceleration. Under empirical hardware profiling, the network exhibits a baseline video memory (VRAM) footprint of 4.2 GB during continuous inference, with the GPU core drawing approximately 145 W. This overhead is primarily introduced by the frequent memory read–write cycles and Memory Access Cost (MAC) inherent to the multi-scale feature aggregating paths within the MLCA block. Combined with the sequential, non-parallelized CPU execution logic of the heuristic SCMCg algorithm, these factors introduce minor processing latencies. While 29 FPS fully satisfies the processing requirements of standard vehicle inspections, executing this pipeline on power-constrained embedded systems (such as NVIDIA Jetson Orin Series edge computers) will inevitably cause a processing speed drop due to the computational delta between server-grade and edge-grade hardware.

To mitigate this hardware performance bottleneck, future edge deployment will necessitate tensor optimization strategies, specifically half-precision (FP16) or integer (INT8) quantization. In practical intelligent transportation infrastructure, compiled TensorRT engine deployment proves that converting the model framework from PyTorch FP32 to TensorRT FP16 effectively halves VRAM utilization and accelerates inference speed by 1.8× to 2.4× while maintaining an identical precision envelope. Conversely, while standard INT8 quantization aggressively strips down latency, it typically induces a catastrophic 5% to 10% degradation in detection mAP due to severe quantization noise in handling structural edges. Given that road sign tracking demands sub-pixel stability to align with absolute GPS topologies, our future work will explicitly bypass naive INT8 post-training quantization, focusing instead on Quantization-Aware Training (QAT) to preserve topological precision. Concurrently, offloading the SCMCg triangulation pipeline onto GPU/CUDA asynchronous compute streams will alleviate CPU-bound latency, guaranteeing robust, low-power edge performance without compromising tracking spatial accuracy.

## 6. Conclusions

This study introduces YOLO-DeepSort, a comprehensive spatio-temporal tracking and detection framework designed for the automated inspection and maintenance of highway infrastructure. By refining the YOLOv9 baseline with MLCA and DualConv modules, the feature extraction capability for occluded and densely packed road signs is measurably improved, while the network parameters are tightly constrained to 21.4 M. To translate localized frame detections into geometrically consistent spatial data, the framework integrates the DeepSort tracking algorithm with a novel Spatial Multivariate Clustering Algorithm with GPS information (SCMCg). This integration successfully eliminates redundant geographic mapping and sustains tracking continuity across dynamic video sequences. Evaluated on a combined dataset comprising the CCTSDB and self-collected national road data, the proposed framework achieved a Precision of 97.8%, an mAP of 91.2%, and a peak tracking Success Rate of 97.3%. The proposed solution establishes an optimal balance between structural lightweighting and spatial robustness, providing a highly reliable methodology for the continuous monitoring and condition assessment of traffic signs. Future engineering iterations will prioritize TensorRT FP16 quantization and GPU-accelerated spatial clustering to bridge the computational gap for robust high-speed edge deployments.

## Figures and Tables

**Table 1:** Optimized hyperparameters.

Hyperparameters	Parameters
lr0	0.0021
lrf	0.017
momentum	0.937
box	0.05
cls	0.746
cls_pw	1.0
obj	0.7
obj_pw	1.0
dfl	0.561
iou_t	0.20
hsv_h	0.011
hsv_s	0.535
hsv_v	0.137
translate	0.124
fl_gamma	0.0

**Table 2:** Quantitative comparison of different tracking and detection models.

Model	Precision	Recall	mAP	MOTA (%)	IDF1 (%)	IDs (↓)	Success Rate	Parameters (M)	GFLOPs	Processing Speed (FPS)
CNN-DeepSort	0.847	0.836	0.866	72.5	70.2	185	0.823	23.5	86.4	35
YOLOv5l-DeepSort [31]	0.919	0.786	0.828	78.4	76.5	142	0.891	46.5	109.1	52
YOLOv7-DeepSort [32]	0.841	0.698	0.774	74.1	72.8	160	0.807	36.9	104.7	55
YOLOv8-DeepSort [5]	0.932	0.791	0.870	81.2	80.1	115	0.904	25.9	78.9	62
CCSPNet-Joint [33]	0.974	0.924	0.958	83.5	82.3	98	0.928	32.1	94.2	42
FRCNN-DeepSort [34]	0.925	0.859	0.843	79.6	78	130	0.904	41.5	210.3	18
MSTSN-DeepSort	0.984	0.983	0.984	77.8	75.4	195	0.953	15.8	32.5	68
ByteTrack	0.965	0.931	0.942	82.5	81.8	70.5	108	0.895	28.3	75
BoT-SORT	0.971	0.945	0.955	84.8	84.1	73.6	82	0.912	31.6	54
YOLO-DeepSort (Ours)	0.978	0.921	0.912	87.4	86.5	42	0.973	21.4	88.5	29

Note: The down arrow (↓) indicates that lower values correspond to better performance.

**Table 3:** Ablation analysis of the structural components within the proposed framework.

Model Modify				Precision	Recall	mAP	MOTA (%)	IDF1 (%)	HOTA (%)	IDs (↓)	Success
YOLO Parameter	MLCA	Dual Conv	SCMCg								
				0.933	0.817	0.831	80.5	79.2	68.3	128	0.907
√				0.945	0.832	0.837	81.1	80.0	69.1	124	0.921
√	√			0.959	0.824	0.894	84.6	83.1	73.5	95	0.929
√	√	√		0.978	0.822	0.912	85.8	84.4	74.8	88	0.958
√	√	√	√	0.978	0.822	0.912	87.4	86.5	77.2	42	0.973

Note: The down arrow (↓) indicates that lower values correspond to better performance. The checkmark "√" indicates that the corresponding module or optimization strategy is integrated into the model.

**Table 4:** Performance comparison of different backend spatial clustering algorithms using an identical YOLO-DeepSort front-end.

Front-End Architecture	Backend Clustering Module	Parameter Configuration	Tracking Success Rate	Processing Speed (FPS)
YOLO-DeepSort	None (Raw Output)	N/A	82.10%	62
YOLO-DeepSort	K-Means	K (dynamically estimated)	85.30%	45
YOLO-DeepSort	DBSCAN	*ε* = 5.0 m, MinPts = 3	89.40%	38
YOLO-DeepSort	SCMCg (Ours)	*α* = 0.5, *β* = 0.5	97.30%	29

## Data Availability

The publicly available CCTSDB dataset analyzed in this study can be found in the open-source repository referenced in the manuscript. The self-collected Ningxia national road dataset presented in this study is available upon reasonable request from the corresponding author. The data are not publicly available due to security and privacy restrictions regarding the geographical coordinate data of the national highway infrastructure.

## Abbreviations

The following abbreviations are used in this manuscript:

CCTSDB	Chinese Traffic Sign Detection Benchmark
CNN	Convolutional Neural Network
DBSCAN	Density-Based Spatial Clustering of Applications with Noise
DFS	Depth-First Search
DT	Delaunay Triangulation
FPS	Frames Per Second
GIS	Geographic Information System
GPS	Global Positioning System
HOTA	Higher Order Tracking Accuracy
IDF1	Identification F1-score
IMU	Inertial Measurement Unit
mAP	mean Average Precision
MLCA	Mixed Local Channel Attention
MOTA	Multiple Object Tracking Accuracy
SCMCg	Spatial Multivariate Clustering Algorithm with GPS information
YOLO	You Only Look Once
